# Association of Stress, Mental Health, and *VEGFR-2* Gene Polymorphisms with Cardiometabolic Risk in Chinese Malaysian Adults

**DOI:** 10.3390/nu11051140

**Published:** 2019-05-22

**Authors:** Roseline W. K. Yap, Mei-Hua Lin, Yoshihiro Shidoji, Wai Sum Yap

**Affiliations:** 1 School of Biosciences, Taylor’s University, Subang Jaya 47500, Selangor, Malaysia; 2 School of Science and Technology/Psychology, Sunway University, Selangor 47500, Malaysia; mhlin@sunway.edu.my; 3 Graduate School of Human Health Science, University of Nagasaki, Nagayo-cho, Nagasaki 851-2195, Japan; shidoji@sun.ac.jp; 4 Faculty of Applied Sciences, UCSI University, Taman Connaught, Kuala Lumpur 56000, Malaysia; wsyap@ucsiuniversity.edu.my

**Keywords:** gene-environment interaction, *VEGFR-2* gene, rs1870377, rs2071559, job stress, mental health, cardiometabolic risk, Chinese Malaysian adults

## Abstract

Gene-environment (G × E) interactions involving job stress and mental health on risk factors of cardiovascular disease (CVD) are minimally explored. This study examined the association and G × E interaction effects of vascular endothelial growth factor receptor-2 (*VEGFR-2*) gene polymorphisms (rs1870377, rs2071559) on cardiometabolic risk in Chinese Malaysian adults. Questionnaires: Job Stress Scale (JSS); Depression, Anxiety, and Stress Scale (DASS-21); and Rhode Island Stress and Coping Inventory (RISCI) were used to measure job stress, mental health, and coping with perceived stress. Cardiometabolic risk parameters were evaluated in plasma and genotyping analysis was performed by real-time polymerase chain reaction. The subjects were 127 Chinese Malaysian adults. The allele frequencies for rs1870377 (A allele and T allele) and rs2071557 (A allele and T allele) polymorphisms were 0.48 and 0.52, and 0.37 and 0.63, respectively. Significant correlations include scores from JSS dimensions with blood glucose (BG) (*p* = 0.025–0.045), DASS-21 dimensions with blood pressure, BMI, and uric acid (*p* = 0.029–0.047), and RISCI with blood pressure and BG (*p* = 0.016–0.049). Significant G × E interactions were obtained for: rs1870377 with stress on total cholesterol (*p* = 0.035), low density lipoprotein cholesterol (*p* = 0.019), and apolipoprotein B100 (*p* = 0.004); and rs2071559 with anxiety on blood pressure (*p* = 0.006–0.045). The significant G × E interactions prompt actions for managing stress and anxiety for the prevention of CVD.

## 1. Introduction

Cardiovascular diseases (CVDs), especially heart attack and stroke, are still the number one killer above any other cause, which affect several countries globally [1]. Similarly, in Malaysia, CVDs contribute to the main cause of death, with a combined total of 20.1% in 2016 from coronary heart disease (CHD) and cerebrovascular disease [2]. In addition, the prevalence of metabolic risk factors of CVD, namely hypertension, diabetes mellitus, hypercholesterolemia, and overweight or obesity, is high among Malaysian adults, with a total of 30.3%, 17.5%, 47.7%, and 47.7%, respectively, in 2015 [3].

The known etiology of CVD includes non-modifiable risk factors (age, sex, family history, and genes) and modifiable, behavioral, and environmental risk factors (unhealthy diet, physical inactivity, tobacco use, and harmful use of alcohol). However, there are two interrelated environmental risk factors—job stress, and mental health, which are minimally explored thus far, and can contribute to CVD. Stress can increase the risk of CVDs via physiological mechanisms, such as increasing pulse rate, blood pressure, and clotting tendencies [4]. In addition, a meta-analysis [5] comprised of European cohort studies has reported that psychosocial stress (job strain) was significantly associated with the risk of CHD. In terms of the relationship between mental health and CVD, depression has been shown to increase the risk of CHD by at least 1.5 times in healthy individuals [6], while anxiety was associated with a more than 50% increase in the incidence of CVD [7].

There are limited data on the prevalence of job stress among working adults in Malaysia. However, an online survey in 2013 conducted by a global workplace provider, Regus, has reported that approximately 70% of the working adults in Malaysia have experienced an increase in stress-related illnesses [8]. Recent surveys conducted in 2017 and 2018 by an insurance company, American International Assurance (AIA) Group Limited, on several factors related to the workplace have shown that more than 50% of working Malaysian adults experienced at least one related stress factor [9,10]. In terms of mental health, the National Health and Morbidity Survey of Malaysia 2015 has also reported a prevalence of around 30% for mental health problems among Malaysian adults aged 16 years and above [3].

Clinical studies on gene-environment (G × E) interaction effect involving candidate genes and the environmental factors (job stress and mental health) of CVD and cardiometabolic risk are limited, especially in Malaysia. However, there are studies involving Chinese populations in China, which showed promising G × E interactions on cardiometabolic risk, such as metabolic syndrome [11]. In terms of suitable candidate genes to represent the genetic factor, our previous study on associations involving single-nucleotide polymorphisms (SNPs) in the *VEGFR-2* gene (rs1870377, rs2071559) has shown significant and promising results on the metabolic risk factors of CVD in Chinese Malaysian adults [12]. However, there is no co-relation in terms of the SNPs in the *VEGFR-2* gene (rs1870377, rs2071559) between Chinese Malaysian population with other populations, such as Han Chinese [13,14,15] and Japanese populations [16], on cardiometabolic risk. The *VEGFR-2* gene SNPs were also shown to be associated with depression [17]. Hence, the aim of this study is to investigate the associations and G × E interaction effect of stress, mental health, and *VEGFR-2* SNPs (rs1870377, rs2071559) on cardiometabolic risk in Chinese Malaysian adults. There are three hypotheses in our study: (1) There will be an interaction between *VEGFR-2* gene SNPs with stress and mental health on cardiometabolic risk. (2) There will be differences between SNPs in the *VEGFR-2* gene on cardiometabolic risk. (3) Psychological factors, such as job stress, mental health of psychological well-being, and coping with perceived stress, are related to cardiometabolic risk. Hypothesis 1 is the primary outcome, while hypotheses 2 and 3 are secondary and tertiary outcomes.

## 2. Materials and Methods

### 2.1. Study Population and Design

The study subjects were Chinese Malaysian adults aged 30–65 years old, and residents of urban Klang Valley. Subjects were recruited based on the inclusion criteria of Malaysian citizenship, full-time working adults, not pregnant, and the offspring of two generations of Chinese ethnic group. Subjects were excluded if they were taking any medications for chronic non-communicable diseases, such as CVD, and for mental health problems, such as depression. The calculation of sample size was based on the formula *n* = Z^2^_1−α/2_ × (p) × (1 − p)/d^2^ [18]. The Z statistic for level of confidence (_1−α/2_) was set at 95% confidence level, so _1−α/2_ = 1.96; expected prevalence (p) was based on the prevalence rate of CVD among Malaysian adults of 32% in 2010 [19], so healthy adults without CVD would be 68%, therefore p = 0.68; and precision (d) was set at 0.10, which is recommended for the prevalence rate of between 10 to 90%. The minimum sample size required from this calculation is 84 subjects. However, based on the major criterion of this study, which is to include only Chinese Malaysian subjects, 30% was added because the percentage of this ethnic group was found to be 28.6%. [20], and an additional 30% was added for incomplete questionnaires or any other study procedures. Hence, the final calculated sample size for this study is 143 subjects. Convenience sampling approach was applied for this observational study. This study obtained Human Ethics Clearance and approval from the Human Ethics Committee of Taylor’s University (HEC/2016/SBS/001). All subjects voluntarily consented to participation in this study.

### 2.2. Health and Lifestyle Information and Determination of Job Stress, Mental Health, and Coping with Perceived Stress

The following socio-demographic, health, and lifestyle information: age, gender, past or current presence of common chronic non-communicable diseases and mental health problems, including the use of medications, exercise patterns, tobacco use, and alcohol consumption were obtained using a standard questionnaire. In addition to the standard questionnaire, three sets of validated questionnaires were used to measure job stress, mental health, and coping with perceived stress, respectively: (1) Job Stress Scale (JSS); (2) Depression, Anxiety, and Stress Scale (DASS-21); and (3) Rhode Island Stress and Coping Inventory (RISCI). The JSS questionnaire [21] is comprised of 13 items to measure the dimensions of time stress and job-related anxiety using a 5-point Likert scale. The total score for each dimension is obtained from summing the related items of the respective dimension. The total score for job stress can also be determined by summing the scores from both dimensions of time stress and job-related anxiety. Higher scores indicate higher levels of time stress, job-related anxiety, and overall job stress. The DASS-21 questionnaire [22] measures emotional states, which are categorized into 3 subscales, namely depression, anxiety, and stress. This questionnaire has 21 items and uses a 4-point Likert scale to construct the total scores for each subscale. Similarly, with JSS questionnaire, higher scores indicate the severity of the emotional states. There are cutoff values for the interpretation of DASS scores into categories from normal to extremely severe for each of the subscales [23]. Hence, the total scores from each of the three components of DASS-21 were divided into two categories, namely “normal” and “mild to extreme severe” (MES) for G × E interaction analysis. Lastly, the RISCI questionnaire [24] uses a 5-item scale to examine the perceived ability to cope independent of any specific stressful situation. The total score is obtained by summing all the items in the scale and a higher total score means better coping with perceived stress. All of the subjects completed the questionnaires on their own and they were assisted by trained research assistants if they had any queries or needed clarification.

### 2.3. Physical Measurements, Biomarkers, and Genotyping

The subjects’ height to the nearest 0.1 cm, body mass index (BMI), and body fat percentage (BFP) were obtained using a portable stadiometer (Seca 213, Seca, Hamburg, Germany) and a body composition monitor (KaradaScan HBF-375, Omron Health Care Co., Ltd., Kyoto, Japan), respectively. The subjects’ systolic (SBP) and diastolic (DBP) blood pressure values were obtained using an automated blood pressure monitor (Omron SEM-1, Omron Health Care Co., Ltd., Kyoto, Japan). Duplicates of all measurements were obtained and the average values were used for data analysis.

A private pathological laboratory (Pathology and Clinical Laboratory (M) Sdn Bhd) was engaged to analyze the values of the following biomarkers: blood glucose (BG); glycated hemoglobin A1c (HbA1c); uric acid (UA); and blood lipid profile (total cholesterol (TC), triglycerides (TG), low-density lipoprotein cholesterol (LDL-C), high-density lipoprotein cholesterol (HDL-C), TC/HDL-C ratio, apolipoproteinA1 (apoA1), and apolipoproteinB100 (apoB100)) using a 10 mL blood sample drawn from each subject by a phlebotomist. In this study, a minimum of 2-hour postprandial blood sample was collected in order to increase the participation and to increase the convenience for the subjects. Studies have also indicated minimal differences in mean blood glucose [25] and blood lipid [26,27,28,29] levels when measured during fasting or in a non-fasted state. In addition, blood lipids (HDL-C, TG, TC/HDL-C ratio, and apoA1) have been shown to predict CVD when measured in a non-fasted state [27]. The use of the HbA1c parameter in our study was to diagnose any abnormalities in blood glucose levels.

DNA samples of all subjects from buccal mucosal cells were obtained using polyester fiber tipped applicator swabs (Falcon; Becton Dickinson and Company, Sparks, MD, USA). DNA extraction and purification procedures were performed using QIAamp DNA Blood Mini kit (Qiagen, Germantown, MD, USA). The Real-time Polymerase Chain Reaction (RT-PCR) system (StepOne™, AppliedBiosystems, Singapore, Singapore) was applied in the genotyping analyses for *VEGFR-2* gene SNPs (rs1870377, rs2071559) using Taqman^®^ GTXpress Master Mix (Applied Biosystems, Foster City, CA, USA) and ready-made TaqMan probes (Taqman SNP Genotyping Assays, Applied Biosystems, Foster City, CA, USA). Genotyping procedures were performed by following the protocol described by the manufacturer.

### 2.4. Statistical Analysis

Statistical analyses were performed using Statistical Package for Social Sciences (SPSS 20.0, IBM SPSS, Armon, NY, USA) software. Normality test using Kolmogorov-Smirnov test was first performed for continuous data. All data for the cardiometabolic risk parameters were in normal distribution except for SBP, BG, HbA1c, TG, and apoA1. SBP, TG, and apoA1 variables were transformed using log10, while non-parametric tests (Mann-Whitney test; Kruskal-Wallis test; and Spearman’s rho correlation test) were applied for BG and HbA1c variables because the data were still not in normal distribution after log10 transformation. The following statistical methods were used to test the respective hypotheses of our study: two-way ANOVA adjusted for the confounding variable, exercise, to determine G × E interactions for Hypothesis 1; one-way analysis of variance (ANOVA) and Kruskal Wallis test were used for Hypothesis 2 on the differences of SNPs in the *VEGFR-2* gene on cardiometabolic risk; and correlation tests (Pearson Correlation and Spearman’s Rho) for Hypothesis 3 on the scores from JSS, DASS-21, and RISCI with cardiometabolic risk. The possible confounding variables, such as age, gender, tobacco use, and alcohol consumption, were excluded in the analysis of G × E interactions because these variables violated the assumptions required to perform two-way ANOVA tests. A probability value of < 0.05 was set as statistically significant.

## 3. Results

### 3.1. Characteristics of the Subjects

The total number of subjects included in this study was 127 Chinese Malaysian adults with more females (64%) than males (36%). Table 1 summarizes the mean and standard deviations of the measured cardiometabolic risk parameters and genetic characteristics of the subjects. The genotype frequencies of all the subjects for the *VEGFR-2* gene SNPs (rs1870377, rs2071559) were: 25% of AA (*n* = 32), 47% of AT (*n* = 59), and 28% of TT (*n* = 36); and 15% of CC (*n* = 19), 45% of CT (*n* = 57), and 40% of TT (*n* = 51), respectively. The genotype frequencies by gender for the *VEGFR-2* gene SNPs (rs1870377, rs2071559) were: 20% of AA (*n* = 16), 49% of AT (*n* = 40), and 31% of TT (*n* = 25); and 17% of CC (*n* = 14), 48% of CT (*n* = 39), and 35% of TT (*n* = 28), respectively, for females. For males, these were: 35% of AA (*n* = 16), 39% of AT (*n* = 18), and 26% of TT (*n* = 12); and 9% of CC (*n* = 4), 39% of CT (*n* = 18), and 52% of TT (*n* = 24), respectively. The genotype SNP sites for the subjects in this study were conformed with the Hardy-Weinberg equilibrium using a web-based tool [30]. The minor allele frequency (MAF) for rs2071559 SNP was similar to the Han Chinese population [31] and consistent with our previous study [12]. However, there was a slight difference in the allele frequency for rs1870377 SNP, in which the A allele and T allele values were 0.48 and 0.52 in the present study compared to 0.52 and 0.48 in our previous study [10] and in the Han Chinese population [32]. This could be due the difference in MAF for rs1870377 SNP between males and females in the present study (Table 1). Table 1 shows that there were significant (*p* < 0.001–0.014) differences in mean by gender for all the cardiometabolic risk parameters measured in this study, except for total cholesterol levels (*p* = 0.08). Overall, males had poorer health status compared to females, and this finding is similar to our previous study involving Chinese Malaysian adults [12].

### 3.2. Associations of VEGFR-2 Gene SNPs (rs1870377, rs2071559) with Cardiometabolic Risk Parameters

The results on the differences in the mean for all the measured cardiometabolic risk parameters by genotype of SNPs in the *VEGFR-2* gene (rs1870377, rs2071559) are presented in Table 2 and Table 3, respectively, with no significant differences (*p* > 0.05). Analysis of genetic associations were also performed separately for males and females. There was only a significant difference in mean (*p* = 0.034) by genotype for rs2071559 SNP on HbA1c levels in females. Females of CC genotype had significantly higher mean HbA1c levels compared to CT genotype subjects. There were no significant genetic associations with cardiometabolic risk parameters in males (*p* > 0.05).

### 3.3. Total Scores from JSS, DASS-21, and RISCI and Correlations with Cardiometabolic Risk Parameters

Three components are assessed from the JSS questionnaire: time stress (JSSS); job anxiety (JSSA); and overall job stress (JSS). This is also similar for the DASS-21 questionnaire, which is used to measure three components: depression (DASSD); anxiety (DASSA); and stress (DASSS). The mean scores ± S.D. obtained from JSS were 20.6 ± 6.51 for time stress, 13.4 ± 3.89 for job-related anxiety, and 34.0 ± 9.88 for overall job stress. The range of scores which can be achieved from the JSS questionnaire are 8–40 for time stress, 5–25 for job-related anxiety, and 13–65 for overall job stress [21]. The mean levels obtained in the present study were all below the midpoint of the maximum scores. The mean scores ± S.D. obtained for DASS on the other hand were 5.75 ± 5.56 for depression, 6.28 ± 5.52 for anxiety and 8.66 ± 6.34 for stress. The scores obtained from the three components of DASS-21 indicate normal levels [23]. Finally, the mean score ± S.D. obtained from the RISCI questionnaire was 18.9 ± 3.10, indicating above average scores for coping with perceived stress, since the range of scores which can be obtained is 5–25 [24]. There were no significant differences in mean scores obtained from JSS, DASS-21, and RISCI questionnaires between males and females (*p* = 0.091–0.867).

Correlation tests were performed for scores from JSS, DASS-21, and RISCI with age and cardiometabolic risk parameters. There were significant correlations between age with time stress (*r* = −0.236; *p* = 0.008), job-related anxiety (*r* = −0.208; *p* = 0.019), and overall job stress (*r* = −0.237; *p* = 0.007) in JSS and coping with perceived stress (*r* = 0.314; *p* < 0.001). Older subjects had lower scores for time stress, job-related anxiety, overall job stress, and better scores in coping with perceived stress. Blood glucose levels, however, were inversely correlated with time stress (*r* = −0.190; *p* = 0.032), job-related anxiety (*r* = −0.179; *p* = 0.045), and overall job stress (*r* = −0.199; *p* = 0.025) in JSS, while positively correlated with coping of perceived stress (*r* = 0.213; *p* = 0.016). In terms of blood pressure, both SBP and DBP were inversely correlated with DASS depression (*r* = −0.186; *p* = 0.036 and *r* = −0.088; *p* = 0.034 respectively), and only DBP was correlated with DASS anxiety (*r* = −0.177; *p* = 0.047). However, both SBP and DBP values were positively correlated with scores for coping with perceived stress (*r* = 0.175; *p* = 0.049 and *r* = 0.186; *p* = 0.037 respectively). Other significant associations include inverse correlations for uric acid levels with DASS anxiety (*r* = −0.180; *p* = 0.043), and BMI with DASS stress (*r* = −0.193; *p* = 0.029). In summary, the results showed that the higher the levels of the cardiometabolic risk parameters, namely BMI, blood pressure, blood glucose, and uric acid, the lower the scores for job stress and mental health. However, better coping with perceived stress was significantly associated with higher levels of blood glucose and blood pressure.

### 3.4. G × E Interactions of VEGFR-2 Gene SNPs (rs1870377, rs2071559) with DASS-21 Scores on Cardiometabolic Risk Parameters 

Reference values for the categories of normal to extreme-severe are only available for scores obtained from DASS-21 questionnaire [23] but not for scores from JSS and RISCI. Hence, G × E interaction analysis involving the *VEGFR-2* gene SNPs (rs1870377, rs2071559) and scores from the three components in DASS-21 was performed on cardiometabolic risk parameters. Significant G × E interaction effects were obtained for rs1870377 and DASS stress on total cholesterol, LDL-C, and apolipoprotein B100 levels (Table 4), and rs2071559 with DASS anxiety on blood pressure levels (Table 5).

It is shown that the combination of TT genotype of rs1870377 and “Mild to Extreme Severe (MES)” category in DASS stress had the highest mean for total cholesterol, LDL-C, and apolipoprotein B100 levels compared to the other five combinations (Table 4). Similarly, for the G × E interaction between rs2071559 and DASS anxiety, the combination of TT genotype of rs2071559 and DASSA “MES” category had the highest mean value for diastolic blood pressure (Table 5).

## 4. Discussion

The increase and high prevalence of cardiometabolic risk, job stress, and mental health in the Malaysian population has prompted the need for a multidisciplinary approach in tackling the mortality and co-morbidities of CVD. The approach of G × E interaction, which can incorporate possible risk factors, could be effective in providing useful information for the prevention and treatment measures of CVD. Hence, this study investigated the associations and interaction effects of *VEGFR-2* gene SNPs with stress and mental health on cardiometabolic risk parameters in Chinese Malaysian adults. In the present study, significant findings on the correlations of job stress, mental health, and coping with perceived stress with several cardiometabolic risk parameters, namely blood pressure, BMI, blood glucose, and uric acid, were obtained. Importantly, significant G × E interactions were obtained for two key cardiometabolic risk parameters, which are blood pressure and blood lipids.

The present study did not obtain significant associations between *VEGFR-2* gene SNPs and any of the cardiometabolic risk parameters in our subjects. These results were different from our previous study in Chinese Malaysian adults, which reported significant differences in means for total cholesterol and HDL-C levels for rs1870377 SNP and LDL-C levels for rs2071559 SNP [12]. The possible explanation could be due to the slight differences in the MAF between males and females, which are shown in our study. Hence, repetition with even distribution of males and females may be required to confirm our findings.

Our investigation has shown significant correlations between the factors of job stress, mental health, and coping with perceived stress with several cardiometabolic risk parameters. The positive associations between job stress and mental health with the risk of CVD have been well established in reviews and meta-analyses [6,7,33]. However, there are limited studies on the association between job stress, mental health, and perceived stress coping with the metabolic risk factors of CVD, namely raised blood pressure, overweight and obesity, hyperglycemia, and hyperlipidemia. Findings from a large sample of 43,593 French adults showed that work stress was associated with higher BMI, blood triglycerides, LDL-C, and lower HDL-C levels in both men and women with no significant associations with blood pressure [34]. It was also reported that work stress was associated with higher blood glucose only in males [34]. Our study, on the other hand, reported that subjects with lower scores for job stress and mental health had higher BMI, blood pressure, blood glucose, and uric acid. The possible explanations could be due to the different populations, small sample size, below average scores or low scores for job stress, and normal scores for mental health obtained in our subjects.

Our study has showed significant G × E interactions for *VEGFR-2* gene SNPs (rs1870377, rs2071559) with stress and anxiety on blood lipids and blood pressure. Both rs1870377 and rs2071559 are functional SNPs, with the former being a missense SNP, which can influence the function of *VEGFR-2* gene in affecting the binding efficiency of *VEGF* to *VEGFR-2*, while the latter is a regulatory SNP, which can affect the expression levels of *VEGFR-2* gene. In a study [15], which investigated on the associations of both *VEGFR-2* gene SNPs (rs1870377, rs2071559) with CHD, the results showed that T-allele of both SNPs had higher message levels of the *VEGFR-2* gene and higher affinity for VEGF, respectively. Hence, the TT genotype of both SNPs may possess higher VEGF activity. We speculate that the “MES” scores for stress and anxiety, indicating higher stress and anxiety than normal, may further enhance the VEGF activity in TT genotype subjects contributing to the significant G × E interaction effects on the blood lipids and blood pressure levels. However, we did not find any similar studies, which could support our speculation.

There are three limitations in the present study. Firstly, the sample size of our study was below our calculated sample size, therefore this may affect the statistical power. Secondly, there was a smaller sample size of male subjects in the study, hence an uneven distribution of males to females was obtained. Hence, the results may be skewed towards the females, who represented 64% of the total sample. Lastly, even though JSS, DASS-21, and RISCI questionnaires are validated questionnaires, the scores obtained are dependent on the honesty of the subjects when completing the questionnaires.

In this study, there were no significant genetic associations of SNPs in the *VEGFR-2* gene (rs1870377, rs2071559) with cardiometabolic risk parameters in our sample of Chinese Malaysian working adults. However, significant associations were obtained for: job stress, job-related anxiety, depression, and coping with perceived stress with blood glucose and blood pressure levels; anxiety with diastolic blood pressure and uric acid levels; and stress with BMI. In addition, this may be the first report that has shown significant results on gene-environment interactions involving SNPs in the *VEGFR-2* gene (rs1870377, rs2071559) with mental health (stress and anxiety) on blood lipids and blood pressure in Chinese Malaysian working adults. The combinations of the TT genotype of rs1870377 and “mild to severe extreme” category in DASS stress had a higher risk of blood lipids, while TT genotype of rs1870377 and “mild to severe extreme” category in DASS anxiety had a higher risk of blood pressure.

Our study has provided an interesting insight that environmental factors, such as job stress and mental health, particularly anxiety, may interact with *VEGFR-2* gene SNPs contributing to an effect on blood lipids and blood pressure, which are metabolic risk factors of CVD. This may result in a higher risk of CVD in the future. Hence, stress management and having a healthy mental state are crucial for the prevention of CVD, as high stress and poor mental health may interact with related genes of the cardiovascular system. Further research is definitely needed, as there are limited studies on such G × E interaction. A larger sample size is recommended to confirm our findings, and we also recommend further investigation on other ethnic groups in Malaysia, including other candidate gene polymorphisms, to curb the high prevalence of CVD affecting Malaysian adults. In conclusion, the findings of this study can contribute in providing evidence on possible G × E interactions involving job stress and mental health, which may increase the risk of CVD in this population.

## Figures and Tables

**Table 1:** Characteristics of the subjects.

Variables	Males (*n* = 46)	Females (*n* = 81)	Total (*n* = 127)
Age ^†^ (years)	40 ± 9	38 ± 8	39 ± 8
Body Mass Index, BMI ^†^ (kg/m^2^)	24.4 ± 2.70 ^a^	22.5 ± 4.28 ^b^	23.2 ± 3.88
Body Fat Percentage, BFP ^†^ (%)	25.2 ± 3.24 ^a^	31.6 ± 5.26 ^b^	29.3 ± 5.56
Systolic Blood Pressure, SBP ^†^ (mmHg)	129 ± 14.5 ^a^	118 ± 17.6 ^b^	122 ± 17.2
Diastolic Blood Pressure, DBP ^†^ (mmHg)	79.4 ± 10.5 ^a^	74.0 ± 11.1 ^b^	75.9 ± 11.2
Blood Glucose, BG ^‡^ (mmol/L)	5.12 ± 1.37 ^a^	4.64 ± 0.46 ^b^	4.82 ± 0.92
HbA1c ^‡^ (mmol/mol)	38.8 ± 9.23 ^a^	35.5 ± 3.37 ^b^	36.7 ± 6.33
Uric acid ^†^ (mmol/L)	0.38 ± 0.07 ^a^	0.29 ± 0.07 ^b^	0.33 ± 0.08
Total cholesterol ^†^ (mmol/L)	5.43 ± 0.78	5.16 ± 0.89	5.26 ± 0.86
Triglycerides ^†^ (mmol/L)	1.63 ± 0.76 ^a^	1.27 ± 0.79 ^b^	1.40 ± 0.79
LDL-C ^†^ (mmol/L)	3.34 ± 0.73 ^a^	2.86 ± 0.87 ^b^	3.03 ± 0.82
HDL-C ^†^ (mmol/L)	1.35 ± 0.24 ^a^	1.73 ± 0.33 ^b^	1.59 ± 0.35
Total cholesterol/HDL-C ratio ^†^	4.13 ± 0.91 ^a^	3.09 ± 0.81 ^b^	3.47 ± 0.99
Apolipoprotein A1 ^†^ (G/L)	1.48 ± 0.11 ^a^	1.58 ± 0.20 ^b^	1.54 ± 0.18
Apolipoprotein B100 ^†^ (G/L)	1.04 ± 0.19 ^a^	0.90 ± 0.21 ^b^	0.95 ± 0.21
rs1870377 (A allele; T allele)	0.54; 0.46	0.44; 0.56	0.48; 0.52
rs2071559 (C allele; T allele)	0.28; 0.72	0.41; 0.59	0.37; 0.63

Glycated hemoglobin A1c (HbA1c), Low-density lipoprotein cholesterol (LDL-C), High-density lipoprotein cholesterol (HDL-C), Gram per liter (G/L). Data are presented in means ± S.D. Analysis performed using student *t*-test ^†^ and Mann-Whitney test ^‡^. Note: ^a,b^: different letters indicate significant difference between groups (*p* < 0.05).

**Table 2:** Values of cardiometabolic risk parameters according to genotype of *VEGFR-2* gene rs1870377 single-nucleotide polymorphism, SNP (*n* = 127).

Variables	rs1870377			*p*-Value
	AA (*n* = 32)	AT (*n* = 59)	TT (*n* = 36)	
BMI ^†^ (kg/m^2^)	23.6 ± 0.66	22.6 ± 0.49	23.7 ± 0.69	0.313
BFP ^†^ (%)	28.5 ± 0.86	29.2 ± 0.77	30.1 ± 0.94	0.490
SBP ^†^ (mmHg)	123 ± 2.41	123 ± 2.49	120 ± 2.83	0.668
DBP ^†^ (mmHg)	76.7 ± 1.77	75.4 ± 1.53	76.2 ± 1.91	0.874
BG ^‡^ (mmol/L)	5.08 ± 0.26	4.79 ± 0.09	4.64 ± 0.09	0.115
HbA1c ^‡^ (mmol/mol)	38.5 ± 1.86	36.4 ± 0.53	35.5 ± 0.64	0.162
Uric acid ^†^ (mmol/L)	0.33 ± 0.01	0.32 ± 0.01	0.33 ± 0.01	0.540
Total cholesterol ^†^ (mmol/L)	5.41 ± 0.15	5.15 ± 0.11	5.29 ± 0.14	0.382
Triglycerides ^†^ (mmol/L)	1.55 ± 0.15	1.24 ± 0.09	1.53 ± 0.15	0.106
LDL-C ^†^ (mmol/L)	3.12 ± 0.15	3.00 ± 0.11	2.99 ± 0.13	0.754
HDL-C ^†^ (mmol/L)	1.58 ± 0.06	1.60 ± 0.04	1.60 ± 0.07	0.944
Total cholesterol/HDL-C ratio ^†^	3.58 ± 0.18	3.38 ± 0.12	3.51 ± 0.17	0.635
Apolipoprotein A1 ^†^ (G/L)	1.56 ± 0.02	1.52 ± 0.02	1.57 ± 0.04	0.837
Apolipoprotein B100 ^†^ (G/L)	1.00 ± 0.04	0.93 ± 0.03	0.96 ± 0.04	0.301

Data are presented in means ± standard error. Analysis performed using one-way ANOVA ^†^ and Kruskal Wallis test ^‡^.

**Table 3:** Values of cardiometabolic risk parameters according to genotype of *VEGFR-2* gene rs2071559 SNP (*n* = 127).

Variables	rs2071559			*p*-Value
	CC (*n* = 19)	CT (*n* = 57)	TT (*n* = 51)	
BMI ^†^ (kg/m^2^)	24.0 ± 1.21	23.0 ± 0.52	23.0 ± 0.46	0.640
BFP ^†^ (%)	30.9 ± 1.52	29.3 ± 0.68	28.7 ± 0.79	0.319
SBP ^†^ (mmHg)	123 ± 2.41	123 ± 2.49	120 ± 2.83	0.588
DBP ^†^ (mmHg)	74.5 ± 2.20	75.8 ± 1.59	76.6 ± 1.52	0.779
BG ^‡^ (mmol/L)	5.08 ± 0.26	4.79 ± 0.09	4.64 ± 0.09	0.107
HbA1c ^‡^ (mmol/mol)	38.5 ± 1.86	36.4 ± 0.53	35.5 ± 0.64	0.092
Uric acid ^†^ (mmol/L)	0.34 ± 0.02	0.31 ± 0.01	0.33 ± 0.01	0.387
Total cholesterol ^†^ (mmol/L)	5.46 ± 0.24	5.25 ± 0.12	5.19 ± 0.11	0.510
Triglycerides ^†^ (mmol/L)	1.55 ± 0.15	1.24 ± 0.09	1.53 ± 0.15	0.270
LDL-C ^†^ (mmol/L)	3.15 ± 0.22	3.03 ± 0.11	2.98 ± 0.11	0.721
HDL-C ^†^ (mmol/L)	1.59 ± 0.08	1.63 ± 0.05	1.56 ± 0.05	0.656
Total cholesterol/HDL-C ratio ^†^	3.63 ± 0.27	3.39 ± 0.13	3.49 ± 0.13	0.639
Apolipoprotein A1 ^†^ (G/L)	1.56 ± 0.02	1.52 ± 0.02	1.57 ± 0.04	0.565
Apolipoprotein B100 ^†^ (G/L)	1.04 ± 0.06	0.93 ± 0.03	0.94 ± 0.03	0.197

Data are presented in means ± standard error. Analysis performed using one-way ANOVA ^†^ and Kruskal Wallis test ^‡^.

**Table 4:** Interaction between genotypes of *VEGFR-2* gene rs1870377 SNP and categories of Depression, Anxiety, and Stress Scale (DASS) for stress component on cardiometabolic risk parameters.

Variable	DASSS	rs1870377	*n*	Mean ± S.E.	P Interaction
BMI (kg/m^2^)	Normal	AA	29	23.6 ± 0.79	0.458
		AT	51	22.9 ± 0.54	
		TT	31	23.7 ± 0.72	
	MSE	AA	3	23.5 ± 2.24	
		AT	8	20.6 ± 1.37	
		TT	5	24.1 ± 1.74	
BFP (%)	Normal	AA	29	28.5 ± 1.04	0.895
		AT	51	29.5 ± 0.79	
		TT	31	30.3 ± 1.01	
	MSE	AA	3	28.1 ± 3.24	
		AT	8	27.2 ± 1.98	
		TT	5	28.9 ± 2.51	
SBP (mmHg)	Normal	AA	29	124 ± 3.24	0.694
		AT	51	123 ± 2.45	
		TT	31	119 ± 3.14	
	MSE	AA	3	116 ± 10.1	
		AT	8	121 ± 6.18	
		TT	5	122 ± 7.81	
DBP (mmHg)	Normal	AA	29	77.0 ± 2.11	0.697
		AT	51	75.7 ± 1.59	
		TT	31	75.8 ± 2.04	
	MSE	AA	3	73.0 ± 6.55	
		AT	8	73.4 ± 4.01	
		TT	5	78.4 ± 5.08	
BG (mmol/L)	Normal	AA	29	5.11 ± 0.17	0.944
		AT	51	4.80 ± 0.13	
		TT	31	4.66 ± 0.17	
	MSE	AA	3	4.77 ± 0.54	
		AT	8	4.69 ± 0.33	
		TT	5	4.50 ± 0.41	
HbA1c (mmol/mol)	Normal	AA	29	38.5 ± 1.18	0.876
		AT	51	36.5 ± 0.89	
		TT	31	35.4 ± 1.14	
	MSE	AA	3	38.3 ± 3.67	
		AT	8	36.0 ± 2.25	
		TT	5	36.4 ± 2.84	
UA (mmol/L)	Normal	AA	29	0.34 ± 0.02	0.835
		AT	51	0.32 ± 0.01	
		TT	31	0.34 ± 0.02	
	MSE	AA	3	0.31 ± 0.05	
		AT	8	0.30 ± 0.03	
		TT	5	0.29 ± 0.04	
* TC (mmol/L)	Normal	AA	29	5.14 ± 0.15	0.035
		AT	51	5.18 ± 0.12	
		TT	31	5.43 ± 0.16	
	MSE	AA	3	5.17 ± 0.49	
		AT	8	4.98 ± 0.30	
		TT	5	6.20 ± 0.38	
TG (mmol/L)	Normal	AA	29	1.56 ± 0.15	0.738
		AT	51	1.23 ± 0.11	
		TT	31	1.49 ± 0.14	
	MSE	AA	3	1.43 ± 0.46	
		AT	8	1.26 ± 0.28	
		TT	5	1.78 ± 0.35	
* LDL-C (mmol/L)	Normal	AA	29	3.17 ± 0.15	0.019
		AT	51	3.03 ± 0.11	
		TT	31	2.85 ± 0.15	
	MSE	AA	3	2.67 ± 0.47	
		AT	8	2.80 ± 0.29	
		TT	5	3.86 ± 0.36	
HDL-C (mmol/L)	Normal	AA	29	1.55 ± 0.07	0.331
		AT	51	1.60 ± 0.05	
		TT	31	1.62 ± 0.06	
	MSE	AA	3	1.85 ± 0.20	
		AT	8	1.61 ± 0.12	
		TT	5	1.52 ± 0.16	
TC/HDL-C ratio	Normal	AA	29	3.66 ± 0.18	0.063
		AT	51	3.39 ± 0.14	
		TT	31	3.38 ± 0.18	
	MSE	AA	3	2.80 ± 0.57	
		AT	8	3.31 ± 0.35	
		TT	5	4.28 ± 0.44	
ApoA1 (G/L)	Normal	AA	29	1.58 ± 0.03	0.536
		AT	51	1.52 ± 0.03	
		TT	31	1.57 ± 0.03	
	MSE	AA	3	1.66 ± 0.10	
		AT	8	1.50 ± 0.06	
		TT	5	1.58 ± 0.08	
* ApoB100 (G/L)	Normal	AA	29	1.01 ± 0.04	0.004
		AT	51	0.93 ± 0.03	
		TT	31	0.91 ± 0.04	
	MSE	AA	3	0.94 ± 0.12	
		AT	8	0.88 ± 0.07	
		TT	5	1.26 ± 0.09	

Depression, Anxiety, and Stress Scale for Stress component (DASSS), Moderate to Extreme Severe (MES). Data are presented in means ± standard error. Analysis performed using two-way ANOVA adjusted for exercise; * *p* < 0.05.

**Table 5:** Interaction between genotypes of *VEGFR-2* gene rs2071559 SNP and categories of DASS for anxiety component on cardiometabolic risk parameters.

Variable	DASSA	rs2071559	*n*	Mean ± S.E.	P Interaction
BMI (kg/m^2^)	Normal	CC	10	23.4 ± 1.24	0.331
		CT	36	23.5 ± 0.65	
		TT	33	22.8 ± 0.68	
	MSE	CC	9	24.6 ± 1.30	
		CT	21	22.2 ± 0.85	
		TT	18	23.5 ± 0.92	
BFP (%)	Normal	CC	10	30.9 ± 1.78	0.988
		CT	36	29.4 ± 0.94	
		TT	33	28.8 ± 0.98	
	MSE	CC	9	31.0 ± 1.87	
		CT	21	29.0 ± 1.23	
		TT	18	28.5 ± 1.33	
* SBP (mmHg)	Normal	CC	10	128 ± 5.37	0.006
		CT	36	125 ± 2.83	
		TT	33	120 ± 2.96	
	MSE	CC	9	123 ± 5.66	
		CT	21	115 ± 3.71	
		TT	18	126 ± 4.01	
* DBP (mmHg)	Normal	CC	10	75.0 ±3.45	0.045
		CT	36	78.5 ± 1.82	
		TT	33	74.2 ± 1.90	
	MSE	CC	9	74.0 ± 3.64	
		CT	21	71.2 ± 2.38	
		TT	18	81.2 ± 2.57	
BG (mmol/L)	Normal	CC	10	4.83 ± 0.30	0.413
		CT	36	4.89 ± 0.16	
		TT	33	4.82 ± 0.16	
	MSE	CC	9	4.70 ± 0.31	
		CT	21	4.57 ± 0.20	
		TT	18	4.98 ± 0.22	
HbA1c (mmol/mol)	Normal	CC	10	37.1 ± 2.01	0.147
		CT	36	37.4 ± 1.06	
		TT	33	36.4 ± 1.11	
	MSE	CC	9	38.2 ± 2.12	
		CT	21	34.5 ± 1.39	
		TT	18	37.5 ± 1.50	
UA (mmol/L)	Normal	CC	10	0.37 ± 0.03	0.45
		CT	36	0.32 ± 0.01	
		TT	33	0.34 ± 0.01	
	MSE	CC	9	0.30 ± 0.03	
		CT	21	0.30±0.02	
		TT	18	0.33 ± 0.02	
TC (mmol/L)	Normal	CC	10	5.71 ± 0.27	0.44
		CT	36	5.23 ± 0.14	
		TT	33	5.23 ± 0.15	
	MSE	CC	9	5.18 ± 0.29	
		CT	21	5.29 ± 0.19	
		TT	18	5.12 ± 0.20	
TG (mmol/L)	Normal	CC	10	1.60 ± 0.25	0.988
		CT	36	1.28 ± 0.13	
		TT	33	1.49 ± 0.14	
	MSE	CC	9	1.57 ± 0.27	
		CT	21	1.32 ± 0.18	
		TT	18	1.36 ± 0.19	
LDL-C (mmol/L)	Normal	CC	10	3.39 ± 0.26	0.643
		CT	36	3.08 ± 0.14	
		TT	33	3.01 ± 0.14	
	MSE	CC	9	2.89 ± 0.28	
		CT	21	2.95 ± 0.18	
		TT	18	2.91 ± 0.19	
HDL-C (mmol/L)	Normal	CC	10	1.60 ± 0.11	0.481
		CT	36	1.56 ± 0.06	
		TT	33	1.55 ± 0.06	
	MSE	CC	9	1.57 ± 0.12	
		CT	21	1.73 ± 0.08	
		TT	18	1.59 ± 0.08	
TC/HDL-C ratio	Normal	CC	10	3.70 ± 0.32	0.981
		CT	36	3.48 ± 0.17	
		TT	33	3.56 ± 0.17	
	MSE	CC	9	3.57 ± 0.33	
		CT	21	3.23 ± 0.22	
		TT	18	3.35 ± 0.24	
ApoA1 (G/L)	Normal	CC	10	1.59 ± 0.06	0.415
		CT	36	1.53 ± 0.03	
		TT	33	1.52 ± 0.03	
	MSE	CC	9	1.52 ± 0.06	
		CT	21	1.59 ± 0.04	
		TT	18	1.53 ± 0.04	
ApoB100 (G/L)	Normal	CC	10	1.08 ± 0.07	0.731
		CT	36	0.94±0.04	
		TT	33	0.95 ± 0.04	
	MSE	CC	9	0.99 ± 0.07	
		CT	21	0.94 ± 0.05	
		TT	18	0.93 ± 0.05	

Depression, Anxiety, and Stress Scale for Anxiety component (DASSA). Data are presented in means ± standard error. Analysis performed using two-way ANOVA adjusted for exercise; * *p* < 0.05.
